# Apathy as a behavioural marker of cognitive impairment in Parkinson’s disease: a longitudinal analysis

**DOI:** 10.1007/s00415-019-09538-z

**Published:** 2019-10-15

**Authors:** Glen P. Martin, Kathryn R. McDonald, David Allsop, Peter J. Diggle, Iracema Leroi

**Affiliations:** 1 Division of Informatics, Imaging and Data Science, Faculty of Biology, Medicine and Health, University of Manchester, Manchester Academic Health Science Centre, Manchester, UK; 2 Division of Neuroscience and Experimental Psychology, University of Manchester, Manchester, UK; 3 Division of Biomedical and Life Sciences, Faculty of Health and Medicine, Lancaster University, Lancaster, UK; 4 Global Brain Health Institute, Trinity College Dublin, Lloyd Building, Dublin 2, Republic of Ireland; 5 Greater Manchester Mental Health Foundation NHS Trust, Manchester, UK

**Keywords:** Parkinson’s disease dementia (PDD), Mild cognitive impairment in Parkinson’s disease (PD-MCI), Biomarker, Apathy, Neuropsychiatric syndrome

## Abstract

**Background:**

Understanding the longitudinal course of non-motor symptoms, and finding markers to predict cognitive decline in Parkinson’s disease (PD), are priorities. Previous work has demonstrated that apathy is one of the only behavioural symptoms that differentiates people with PD and intact cognition from those with mild cognitive impairment (MCI-PD). Other psychiatric symptoms emerge as dementia in PD develops.

**Objective:**

We explored statistical models of longitudinal change to detect apathy as a behavioural predictor of cognitive decline in PD.

**Methods:**

We followed 104 people with PD intermittently over 2 years, undertaking a variety of motor, behavioural and cognitive measures. We applied a linear mixed effects model to explore behavioural factors associated with cognitive change over time. Our approach goes beyond conventional modelling based on a random-intercept and slope approach, and can be used to examine the variability in measures within individuals over time.

**Results:**

Global cognitive scores worsened during the two-year follow-up, whereas the longitudinal evolution of self-rated apathy scores and other behavioural measures was negligible. Level of apathy was negatively (− 0.598) correlated with level of cognitive impairment and participants with higher than average apathy scores at baseline also had poorer cognition. The model indicated that departure from the mean apathy score at any point in time was mirrored by a corresponding departure from average global cognitive score.

**Conclusion:**

High levels of apathy are predictive of negative cognitive and behavioural outcomes over time, suggesting that apathy may be a behavioural indicator of early cognitive decline. This has clinical and prognostic implications.

**Electronic supplementary material:**

The online version of this article (10.1007/s00415-019-09538-z) contains supplementary material, which is available to authorized users.

## Introduction

The syndrome of mild cognitive impairment in Parkinson’s disease (PD-MCI) occurs in approximately a quarter of people with PD [3, 57]. The most common impairment is that of memory, closely followed by impaired visuospatial function, then attention/executive ability [3]. PD-MCI is considered a harbinger of PDD [34, 57], with an annual conversion rate from PD-MCI to PDD of 11% [34]. While it has been proposed that PD-MCI may remain stable, progress to PDD or revert back to PD with intact cognition [31], a recent study found that those who did revert back to having no cognitive impairment were still at higher risk of developing PD-MCI or PDD in the future [41].

Dementia in PD (PDD) is characterised by deterioration in memory, attention, visuospatial functions, executive functions and the development of behavioural and psychiatric symptoms, such as anxiety, hallucinations and apathy [25, 30]. The impact of PDD, compared to mild cognitive impairment in PD (PD-MCI) is significant, with a marked decrease in functional ability and quality of life and corresponding increase in care partner burden as PDD emerges [51–54]. The prevalence of PDD has been predicted to triple by 2060 [76], underscoring the need to understand the evolution of cognitive impairment in PDD over time and to ascertain the risk factors for developing PDD.

The main risk factors for developing PD-MCI and PDD include older age, akinetic-rigid forms of PD [7, 25], increased disease severity and duration of motor symptoms [57]. Hallucinations [1], REM sleep behaviour disorder [7] and neuropsychiatric syndromes [71] also play a role. A growing number of cohorts of people with PD, e.g. the Parkinson’ progression markers initiative (PPMI, [88]; ICICLE-PD [93]; and the Oxford Parkinson’s Disease Discovery Cohort [37], are exploring the associations of these and other risk factors with the emergence of cognitive decline and dementia in PD. These studies, and others, have identified promising biomarkers of cognitive decline at least two years following a new diagnosis of PD, falling into several categories [42]. These include (1) proteinopathies and fluid biomarkers, such as CSF amyloid *β* (Aβ_42,_ to *t*-tau ratio), apolipoprotein E, and genetic markers COMT val/val and BDNF val/val; (2) neurodegenerative markers, such as decreased global brain volume, a spatial pattern of atrophy resembling Alzheimer’s disease at disease onset, and reduced fractional anisotropy (a measure of structural integrity) using diffusion tensor MRI; (3) neurotransmitter deficiency, such as decreased caudate uptake on dopamine transporter imaging scan; and iv) abnormalities in function and behaviour, including anosmia and REM sleep behaviour disorder [15, 61, 77, 87]. Finally, it has been suggested that, with the correct equipment and methodology, biomarkers may also be detected in the eye [9].

However, identifying biomarkers may be a challenge, given that there may be multiple reasons for cognitive deficits in PD [31]. Nonetheless, it is imperative to find ways to alert clinicians to the onset of cognitive impairment and/or dementia in PD. Thus, the concept of a “pre-PD-MCI” stage is now of interest [31]. Cognitive impairment and behavioural symptoms in PD are closely linked [2, 51–54], with depression, apathy, anxiety and hallucinations representing the most common manifestations [2]. The early onset of hallucinations in PD predicts progression to dementia [1], possibly related to amyloid pathology. In general, more advanced PD and more severe dementia in PD are associated with greater behavioural complications [2]. Thus, there is potential for behavioural markers to be used alongside fluid, neuroimaging and other biomarkers as predictors or indicators of cognitive impairment.

An increasingly recognised link between behavioural change and cognitive impairment in PD is the syndrome of apathy. Apathy can occur in a number of neurodegenerative conditions and psychiatric disorders [38], including Huntington’s disease [86], Alzheimer’s disease [96], fronto-temporal dementia [16], schizophrenia [94] and major depressive disorder [95]. Characterised by reduced motivation [48] apathy is observed as a reduction in voluntary, goal-directed behaviours [56]. There is now consensus between the recently revised diagnostic criteria [72] and the findings from PD [5, 66, 79] and non-PD research [8], with the loss of motivation extending to cognitive, behavioural, emotional and social domains. In the case of PD, apathy may be a consequence of serotonergic depletion [12, 58].

PD apathy is associated with older age, increased levels of depression and lower dopamine agonist use [51–54], is detrimental to disability and health-related quality of life [50] and is associated with increased caregiver burden [51–54]. People with PD and apathy are poorer at processing of certain emotions (fear, disgust and happiness) than with those with PD and no apathy [33], and emotional blunting in PD apathy is associated with poorer quality of life and increased caregiver burden [55].

In cross-sectional studies, apathy has been shown to be the only behavioural symptom to distinguish people with PD-MCI from people with PDD [51–54], or PD and intact cognition [17]. In early stage, untreated PD, the presence of apathy is associated with more severe motor symptoms and poorer cognition [22]. Upon finding five phenotypes of cognitive impairment in PD, Dujardin et al. [23] demonstrated that increased apathy was associated with the three cognitive phenotypes. Longitudinal follow-up of people with PD shows that those with apathy have a higher conversion rate from MCI to PDD than those without apathy [24]. Additionally, apathy is associated with executive dysfunction [63, 75]. Thus, the development of apathy may be a harbinger of dementia [23, 24]. Understanding the longitudinal trajectory and relationship between apathy and cognitive impairment in PD is important; the ability to predict the onset of, and the decline into, PDD foster early detection and intervention.

The objective of this study was to explore the natural pattern of progression of cognitive and behavioural syndromes in PD, and to detect possible behavioural predictors of cognitive decline. Our hypothesis was that the syndrome of apathy would emerge as the strongest candidate predicting change in cognition. Most studies with PD cohorts do not use random effects models. Of those that do, traditional statistical methods of longitudinal analysis involving the random intercept and slope model have usually been applied [6, 69]. However, the assumption that straight lines (i.e. random slopes) can approximate individual random effect trajectories is implausible. Thus, to allow flexibility in our exploration of individual trajectories and potential for non-linear/quadratic associations of cognition/behaviour syndromes, we went beyond the standard random intercept and slope model. Instead, we used longitudinal models that included components to reflect random effects, serial correlation and measurement error. Such methods allowed us to account for natural variability and fluctuation of both the behavioural and cognitive syndrome over time, and thus investigate our hypothesis. To do this, we derived and applied a bespoke joint multivariate linear mixed effects model to explore behavioural factors associated with cognitive change over time [21]. As far as we are aware, this is a novel approach in exploring cognition and behaviour in a PD cohort.

## Methods

### Study design

This was a single centre, non-interventional prospective observational cohort study of people with PD in the north of England. We collected baseline and follow-up visit data over a two-year period with a maximum of five visits per participant. This cohort was embedded within a parent cohort of 204 participants with PD who were being investigated for potential blood biomarkers of PD progression [29]. Of these, *n* = 104 received additional cognitive and behavioural assessments on a repeated basis and were included in the current analysis. We assessed all for capacity to consent to the study and signed informed consent. The study was approved by the South Manchester Research Ethics Committee and was conducted according to standards set by World Medical Association’s Declaration of Helsinki [11].

### Participants

Participants were people with a PD recruited from secondary-care neurological services of the National Health Service (NHS) in the northwest of England. The diagnosis of PD was based on the UK Queen’s Square Brain Bank diagnostic criteria for PD [49], which was made by experienced movement disorder specialists in the respective services. All participants were in disease stage 1 or 2, as per the Hoehn and Yahr scale. Other inclusion criteria were: (1) adults with no upper age cut-off; (2) living at home in the community; (3) capacity to provide consent to participate in the study; and (4) ability to speak and understand English. Any person with a medical, psychiatric or cognitive illness severe enough to interfere with study procedures was not included in the study.

### Measures of clinical variables

At each visit, participants undertook a variety of motor, behavioural and cognitive measures. To assess cognition, we used a general measure of global cognition, the Mini-mental State Exam (MMSE, Folstein, [28]), and a PD-specific measure, the Parkinson’s Disease Cognitive Rating Scale (PD-CRS, [65]). The PD-CRS is a comprehensive cognitive rating battery specifically designed for PD, that can distinguish PD-MCI from PD with intact cognition [43], with validated cut-off scores for PD-MCI [45]. It comprises nine tasks, representing two sub-domains: (1) frontal subcortical tasks (sustained attention, working memory, alternating and action verbal fluency, clock drawing, immediate and delayed free recall verbal memory); and (2) posterior cortical tasks (confrontation naming and clock copying). The PD-CRS can reflect global cognition with a total score, or as ‘subcortical’ and ‘cortical’ profiles, facilitating assessment of the progression to PDD [64].

We used the Movement Disorder Society Unified Parkinson’s Disease Rating Scale part III to assess motor severity [30]. Stage of disease was categorised according to the Hoehn-Yahr scale (H & Y, [27, 35]). Behavioural measures were the informant-rated 12-item neuropsychiatric inventory (NPI-12; [18], the Epworth Sleepiness Scale (ESS; [40]), and self-rated measures of depression, anxiety (Hospital Anxiety and Depression Scale; HADS; [97] and apathy (Apathy Scale; [83]). The Apathy Scale is a self-rated scale consisting of 14 items each of which can be rated on a 4-point Likert Scale. It was specifically adapted from the original Apathy Evaluation Scale (AES; [60]) for use in PD. On this scale, clinically significant apathy can be defined by a score of ≥ 14 (range 0–42) with a sensitivity of 66% and specificity of 100% [83]. It has good face validity, internal consistency, inter-rater and test–retest reliability [48] and is one of the tools recommended for use with PD by the Movement Disorder Society Task Force for use in PD [48]. The NPI is a validated informant-rated scale that assesses 12 domains of behavioural disturbance. It has been used extensively and shown to be valid in PD populations both with and without dementia [2, 4].

To assess quality of life, we used the Parkinson’s Disease Questionnaire 39 (PDQ-39; [39], the EuroQoL Visual Analogue Scale, and the EuroQoL Quality of Life Questionnaire [26]. Finally, functional ability was assessed using the Schwab & England Daily Living Scale [78]. Levodopa daily equivalent dose (LEDD) was calculated according to a recommended formula for total dopaminergic replacement as well as for dopamine agonists only [85].

### Study procedures

Data collection took place in each participant’s home every four to 6 months for 24 months per participant, following the baseline visit. Trained research nurses with extensive experience in PD-specific rating scales undertook the assessments. The assessments were undertaken at the same time each visit, and at least 45–60 min after the participant’s regular PD medication dose. Each participant had a caregiver or informant who knew him or her well, had contact at least once a week, and could provide information on the participant’s behaviour. As this was an observational study, participants were free to attend their regular clinic appointments and receive ‘care as usual’, which included medication changes and other interventions for PD-related symptoms, including referrals to occupational therapists and physiotherapists, and to PD nurse specialists.

### Withdrawal criteria

We assessed participants before each study visit to ensure that they were still able to make informed decisions about having their data collected. If the participant lacked capacity to continue in the study, we nominated a consultee to determine continued participation. Participants who moved into institutionalised care during the study period were withdrawn.

### Statistical analysis

Throughout this study, we defined ‘time zero’ as the date of the first visit in which the cognitive and behavioural assessments were initiated for each of the 104 participants included in this analysis. This time point does not necessarily coincide with their first visit within the parent cohort, since some participants had already enrolled prior to the additional measures being collected. Follow-up time was defined as the number of weeks since time zero.

Our primary outcome measures were PD-CRS (overall score), self-rated Apathy Scale and the apathy sub-scale of NPI (frequency × severity). We modelled both the PD-CRS and self-rated Apathy scales as continuous outcomes, while the NPI apathy sub-score was modelled as a binary variable indicating a frequency × severity score greater than 0. We used linear mixed effects models to model the longitudinal profile of the continuous outcomes. These models included a random intercept, a zero-mean Gaussian stochastic process with an exponential correlation function, and measurement error (see online supplementary methods for mathematical details). To model the apathy sub-scale of NPI (binary indicator of frequency × severity > 0), we used a Binomial generalised linear mixed model with logit link; again, the model included a random intercept and a zero-mean Gaussian stochastic process with an exponential correlation function. We compared the empirical and fitted variograms, where appropriate, to diagnose the fit of our longitudinal models for each outcome. At each stage, the assumptions of the linear mixed effects models were checked by examining the distributions of the residuals.

We then applied multivariate joint longitudinal models to investigate potential relationships between the joint evolutions of the self-rated Apathy Scale and PD-CRS, as per our primary hypothesis of interest. We undertook joint longitudinal modelling of these continuous outcomes by imposing two different assumptions of the random effects joint distribution. Firstly, we assumed that the random intercepts of both outcomes would be correlated. Secondly, we assumed a priori that previous realisations of the self-rated Apathy Scale could be predictive of future realisations of PD-CRS; hence, we extended the correlated random intercept model to also include a distributed lag with directed dependence between the serial correlation terms (see the online supplementary methods for mathematical details). To supplement this analysis, we investigated potential relationships between the NPI apathy sub-score (binary indicator of severity × frequency > 0) and the PD-CRS. Here, we assumed that the random intercepts of both measures would be correlated. Due to the intractability of a joint Binomial and Gaussian likelihood, such a model was fitted using Markov Chain Monte Carlo (MCMC).

All models (both univariate and multivariate) included the following covariates: follow-up time, mean-centred baseline age (at time zero), disease duration at first visit, gender, HY score, indication of working status (at time zero), levodopa daily equivalent dose (LEDD), and an interaction term between baseline age and follow-up time.

As sensitivity analysis, we performed all the aforementioned modelling steps but excluding any observation where the PD-CRS total was below 65 [65]. This was done to capture the possibility that participants with severe cognitive impairment might not be able to accurately score their apathy levels or allow caregivers to differentiate perceived apathy from cognitive impairment and its implications.

We used R version 3.5.1 [70] for all statistical analyses. Data manipulation was undertaken using the ‘tidyverse’ suite of packages [89], with graphical plots made using the ‘ggplot2’ package [90]. Univariate longitudinal models for the continuous outcomes (i.e. PD-CRS and Apathy Scale) were fitted using the ‘lmenssp’ package [10], while the code for the joint modelling of the Apathy Scale and the PD-CRS was written by the authors (avaliable on request). We fitted the generalised linear mixed models using the ‘rstan’ package [81] for both the univariate and multivariate analyses of the NPI apathy domain (i.e. Binomial models), code for which was written by the authors.

## Results

### Participant Baseline Characteristics

The 104 participants had a median follow-up time of 58.1 (min = 0, max = 107) weeks, over a median of 3 (min = 1, max = 5) visits. We have presented the baseline characteristics in Table 1. The majority of participants in our sub-study were male (65.4%). The median age at time of first visit in our study was 68 (min = 27, max = 88) years. The median age at onset of PD symptoms was 62 (min = 26, max = 84) years. The HY score at baseline ranged from 1 (26%) to 2 (44.2%), with only 30.8% of participants working at the start of the study. Twenty-seven participants (26%) reported a family history of PD. Within the cohort, there was a median of 4 (min = 1, max = 16) years between diagnosis and each patient’s first visit. The median LEDD was 382.0, with an inter-quartile range of 192.8–643.0.

**Table 1:** Demographic and baseline characteristics of participants with PD in the cohort (n = 104)

Baseline demographic		Summary
Gender	Female, *n* (%)	36 (34.6%)
	Male, *n* (%)	68 (65.4%)
Hoehn and Yahr scale	1, *n* (%)	27 (26.0%)
	1.5, *n* (%)	31 (29.8%)
	2, *n* (%)	46 (44.2%)
Family history of PD	No, *n* (%)	55 (52.9%)
	Yes, *n* (%)	27 (26.0%)
	Unknown/Patient Unsure, *n* (%)	22 (21.2%)
Working at start of study	No, *n* (%)	72 (69.2%)
	Yes, *n* (%)	32 (30.8%)
Age (years)	at PD onset, median (IQR) [min, max]	62 (55, 69) [26, 84]
	at first visit median (IQR) [min, max]	68 (61, 75) [27, 88]
Disease duration at first visit (years), median (IQR) [min, max]		4 (3, 7) [1, 16]
Levodopa daily equivalent dose, median (IQR) [min, max]		382.0 (192.8, 643.0) [0, 1447]
Baseline variables (as recorded at first Visit)		Summary
Cognition	MMSE, median (IQR) [min, max]	29 (28, 30) [22, 30]
	PD-CRS Total Score, median (IQR) [min, max]	88 (79, 98) [19, 124]
	PD-CRS Subcortical, median (IQR) [min, max]	58.5 (51, 68.5) [17, 94]
	PD-CRS Cortical, median (IQR) [min, max]	29 (28, 30) [0, 30]
Function	Schwab-England score, median (IQR) [min, max]	90 (80, 90) [40, 100]
	EQ-VAS, median (IQR) [min, max]	80 (68.75, 88.5) [30, 100]
	EQ-5D, median (IQR) [min, max]	7 (6, 9) [5, 11]
	PDQ-39 Domain 1, median (IQR) [min, max]	6 (2, 13) [0, 40]
	PDQ-39 Domain 2, median (IQR) [min, max]	4 (2, 8) [0, 21]
	PDQ-39 Domain 3, median (IQR) [min, max]	2 (0, 5) [0, 19]
	PDQ-39 Domain 4, median (IQR) [min, max]	1 (0, 3) [0, 11]
	PDQ-39 Domain 5, median (IQR) [min, max]	0 (0, 0) [0, 5]
	PDQ-39 Domain 6, median (IQR) [min, max]	3 (1, 4.25) [0, 9]
	PDQ-39 Domain 7, median (IQR) [min, max]	1 (0, 1) [0, 8]
	PDQ-39 Domain 8, median (IQR) [min, max]	2 (1, 4) [0, 11]
Motor Score	UPDRS, median (IQR) [min, max]	41.5 (29, 56) [10, 114]
Neuropsychiatric symptoms	NPI total*, median (IQR) [min, max]	3 (1, 9) [0, 46]
	NPI total* ≥ 4, *n* (%)	51 (49.0%)
	NPI total* > 0, *n* (%)	80 (76.9%)
	HADS anxiety, median (IQR) [min, max]	4.5 (3, 7) [0, 17]
	HADS depression, median (IQR) [min, max]	3 (2, 6) [0, 17]
Apathy	Apathy self-rated, median (IQR) [min, max]	10.5 (7, 14) [0, 29]
	NPI apathy sub-score*, median (IQR) [min, max]	0 (0, 0) [0,8]
	NPI apathy sub-score* > 0, *n* (%)	24 (23.1%)
	NPI apathy sub-score* ≥ 4, *n* (%)	6 (5.77%)
Sleep	Epworth Sleepiness Scale, median (IQR) [min, max]	8 (4, 11) [1, 18]
	NPI sleep sub-score*, median (IQR) [min, max]	0 (0, 2.25) [0, 9]
	NPI sleep sub-score* > 0, *n* (%)	39 (37.5%)
	NPI apathy sub-score* ≥ 4, *n* (%)	18 (17.3%)

*PD*-*CRS* Addenbrooke’s Cognitive Evaluation, *EQ* EuroQoL-5D index or visual analogue scale (VAS), *HADS* Hospital Anxiety and Depression Scale, IQR interquartile range, *MMSE* Mini-mental State Exam, *NPI* Neuropsychiatric Inventory, *PDQ*-*39* Parkinson’s Disease Questionnaire, *UPDRS* Unified Parkinson’s Disease Rating Scale, *PD* Parkinson’s Disease

*NPI mean domain score: frequency × severity

### Participant Clinical Profile at Baseline and Follow-up

Baseline descriptors for motor, cognitive, and behavioural ratings are shown in Table 1. Briefly, the median UPDRS at first visit was 42.5 (min = 10, max = 114), and the median Schwab-England score for function was 90 (min = 40, max = 100) at first visit. The baseline distribution of the other functional measures (EQ-VAS, EQ-5D and PDQ-39) showed similar levels of quality of life (Table 1). For cognition, the median baseline PD-CRS total was 88 (min = 19, max = 124); similarly, the subcortical domain of PD-CRS ranged from 17 to 94 (median 58.5) at baseline, and the cortical domain ranged from 0 to 30 (median 29) at baseline (Table 1). At first visit, 21 (20.19%) participants had a PD-CRS between 65 and 82 (PD-MCI), while 9 (8.65%) had a PD-CRS less than 65 for PDD [65]. Out of the 95 (i.e. 104-9) participants who had a PD-CRS total ≥ 65 at first visit, 12 (12.63%) went onto have at least one PD-CRS total score of less than 65 during their follow-up, with a median time to said observation of 28.6 weeks. The median MMSE at baseline was 29 (min = 22, max = 30).

Apathy, our behavioural syndrome of primary interest, was self-rated at a median of 10.5 (min = 0, max = 29) at first visit; *n* = 28 (26.9%) scored above the ‘clinically significant’ threshold for self-rated apathy of 14 points at first visit [33]. Out of the 76 (i.e. 104-28) participants who were below the ‘clinically significant’ threshold for self-rated apathy of 14 points at first visit, 22 (28.9%) went onto have at least one self-rated apathy score above the ‘clinically significant’ 14 points during their follow-up, with a median time to said observation of 32.7 weeks. Informant-rated apathy, as assessed by the NPI apathy domain, ranged between 0 and 9 (medium 0) at first visit, with *n* = 24 (23.1%) and *n* = 6 (5.77%) scoring > 0 and ≥ 4, respectively at baseline (Table 1).

The median (min, max) baseline score for NPI-12 was 3 (0, 46), and 80 participants (76.9%) endorsed at least one of the twelve NPI domains at baseline, 51 (49%) of whom satisfied the ‘clinically significant’ threshold of at least 4 (frequency × severity) (Table 1). At baseline, the most commonly endorsed NPI domains were dysphoria/depression (*n* = 40; 38.5% participants), anxiety (*n* = 40; 38.5%) and night-time behavioural disturbances (*n* = 39; 37.5%), while disinhibition was the least endorsed NPI domain at baseline (*n* = 1; 0.01%). The cohort had a median baseline of 4.5 (min = 0, max = 17) points on the HADS anxiety scale, with *n* = 16 (15.4%) scoring in the mild range (8–11 points), *n* = 4 (3.85%) scoring in the moderate range (12–14 points), and *n* = 3 (2.88%) scoring in the severe range (> 14 points) at first visit. Similarly, the median HADS depression score at first visit was 3 (min = 0, max = 17), with 11 (10.6%), 1 (0.96%) and 1 (0.96%) participants scoring mild, moderate and severe HADS depression, respectively (based on the same cut-offs as HADS anxiety). This level of anxiety and depression was also reflected in the informant-reported anxiety and depression domains of NPI, with *n* = 11 (10.6%) and *n* = 6 (5.8%) scoring in the ‘clinically significant’ range (frequency × severity ≥ 4) for NPI anxiety and depression, respectively. Finally, n = 35 (33.7%) participants had a clinically significant excessive daytime sleepiness at first visit (ESS ≥ 10 [40]), with a median at first visit of 8 (min = 1, max = 18). The median NPI sleep domain at first visit was 0 (min = 0, max = 9), with n = 18 (17.3%) having a ‘clinically significant’ NPI sleep domain (frequency × severity ≥ 4) (Table 1).

**Fig. 1:**
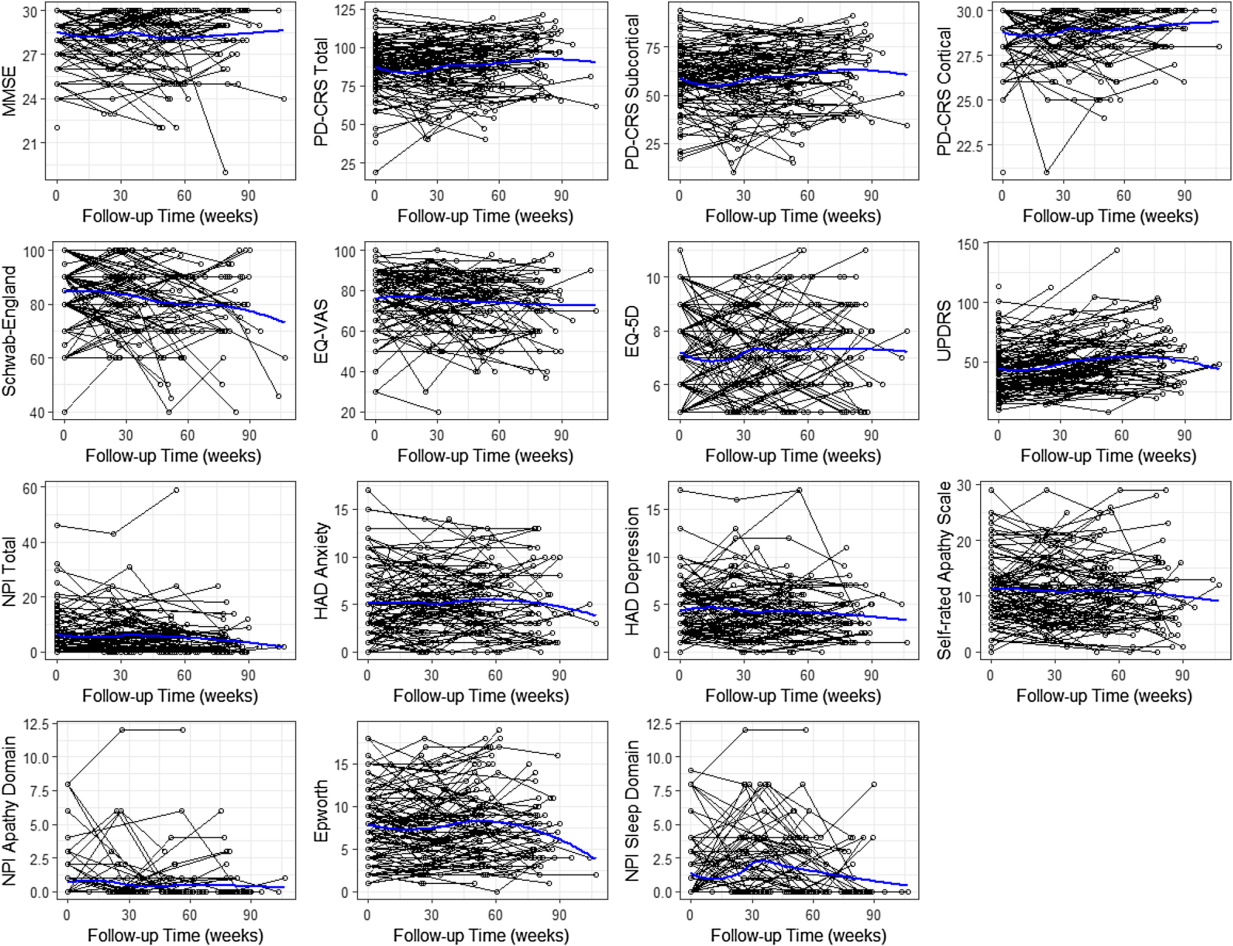
Time-plot showing the temporal evolution of each scale. The blue line shows the average smooth through the points (loess smoother)

Figure 1 and Supplementary Fig. 1 show how these clinical profiles changed through time in follow-up. Additionally, Supplementary Table 1 shows the clinical characteristics of each measure at the time of each participant’s final visit in the study. In general, the clinical characteristics were similar to those described above, with most showing minimal-to-small change through time (Fig. 1).

### Changes in cognition through time

**Fig. 2:**
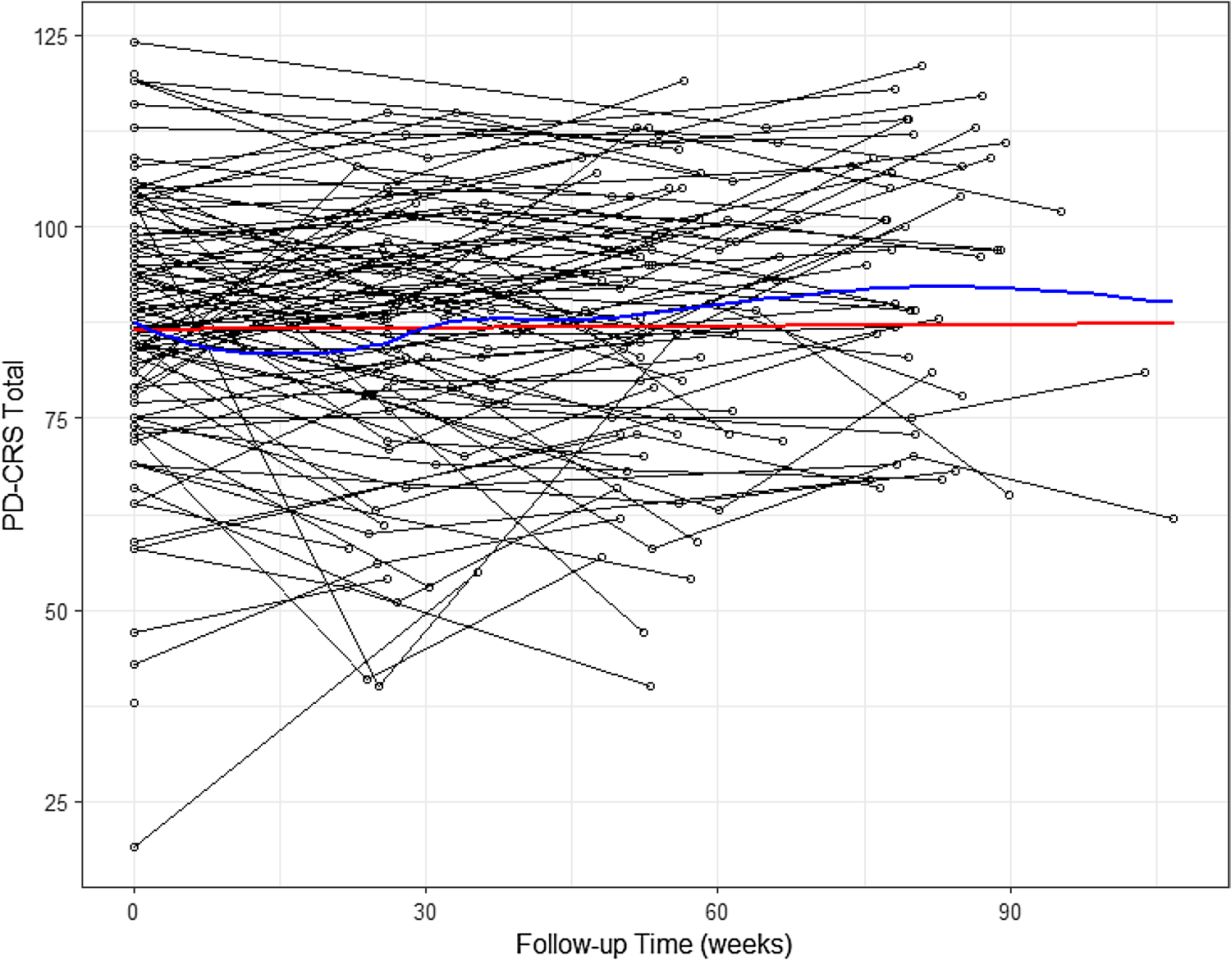
Time-plot of PD-CRS Total. The blue line shows the average smooth through the points (loess smoother), while the red line depicts the mean fitted profile from the univariate model

There was large variability in PD-CRS total scores both within and between participants over time (Fig. 2). However, on average there was only minimal longitudinal change in PD-CRS score. The univariate longitudinal model for total PD-CRS demonstrated that, on average, there was no significant change with follow-up, but baseline age was significantly associated with lower (more impaired) PD-CRS (Table 2). Comparing both the mean fitted response of this model (Fig. 2) and the fitted variogram (Supplementary Fig. 2) with the empirical counterparts demonstrated a reasonable fit of this model to the data, suggesting that our proposed covariance structure for the random effects was reasonable. We observed similar patterns of temporal changes in cognition in both the MMSE, and the subcortical and cortical domains of PD-CRS (Fig. 1). Indeed, univariate longitudinal models of PD-CRS subcortical and PD-CRS cortical demonstrated that cognition did not significantly change with time, but baseline age was associated with worse cognition.

**Table 2:** Fixed-effect parameter estimates and 95% confidence intervals (CI) for the random intercept and serial correlation model for PD-CRS

Variable	Estimate (SE)	95% CI
Intercept	85.03 (4.358)	(76.48, 93.57)
Follow-up time (weeks)	0.007 (0.019)	(− 0.031, 0.044)
Follow-up time × mean-centred baseline age	− 0.002 (0.002)	(− 0.005, 0.002)
Mean-centred baseline age (years)	− 0.767 (0.171)	(− 1.102, − 0.432)
Disease duration at first visit (years)	0.466 (0.455)	(− 0.427, 1.359)
Gender (male vs. female)	− 2.334 (2.910)	(− 8.037, 3.369)
HY Score (< 2 vs 2)	1.056 (2.904)	(− 4.637, 6.748)
Working at start of study (yes vs no)	1.048 (3.408)	(− 5.630, 7.727)
LEDD	0.000 (0.005)	(− 0.010, 0.010)

### Changes in apathy through time

**Fig. 3:**
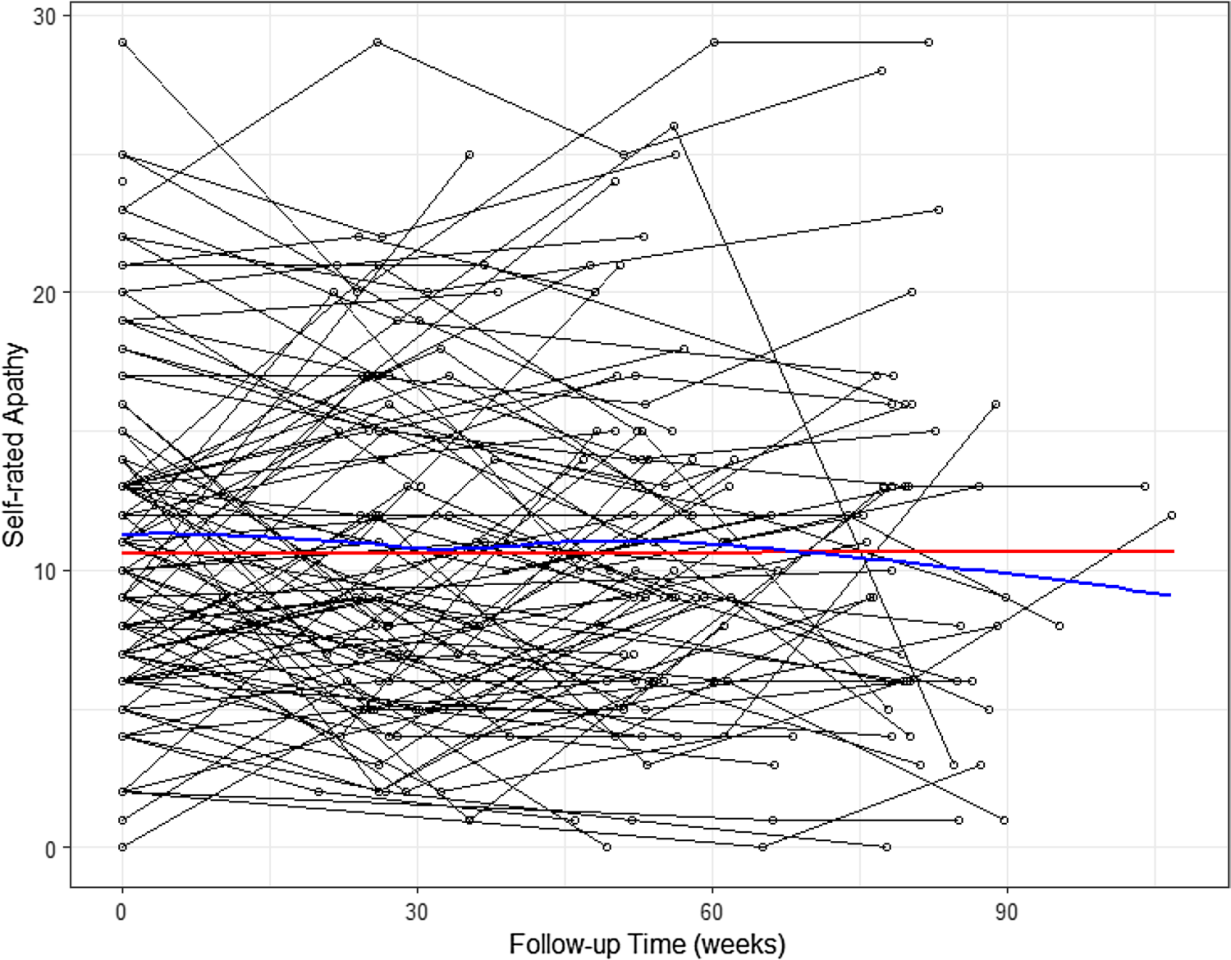
Time-plot of self-rated Apathy Scale. The blue line shows the average smooth through the points (loess smoother), while the red line depicts the mean fitted profile from the univariate model

The longitudinal profile of the self-rated Apathy Scale showed wide variation between participants (Fig. 3), with no apparent trend with increasing follow-up. The fixed-effect parameter estimates from the univariate model of the Apathy Scale in Table 3 highlighted that, on average, there was negligible longitudinal evolution of the self-rated apathy score; however, there was a significant interaction between follow-up time and (mean centred) baseline age. Specifically, a one-year increase in baseline age (over the study average of 67.5 years) increased the association of follow-up time with (more impaired) Apathy Scale scores by 0.003 (95% CI: 0.001, 0.004) points. The mean fitted response line (Fig. 3) and the fitted variogram (Supplementary Fig. 3) indicated a good fit to the data. Similar results were found when modelling the informant-rated apathy as measured with the apathy sub-scale of the NPI (Supplementary Table 2).

**Table 3:** Fixed-effect parameter estimates and 95% confidence intervals (CI) for the random intercept and serial correlation model for self-rated Apathy Scale

Variable	Estimate (SE)	95% CI
Intercept	11.18 (1.654)	(7.937, 14.42)
Follow-up time (weeks)	0.002 (0.007)	(− 0.013, 0.016)
Follow-up time × mean-centred baseline age	0.003 (0.001)	(0.001, 0.004)
Mean-centred baseline age (years)	0.019 (0.065)	(− 0.109, 0.146)
Disease duration at first visit (years)	− 0.086 (0.173)	(− 0.425, 0.252)
Gender (male vs. female)	− 0.262 (1.104)	(− 2.426, 1.902)
HY Score (< 2 vs 2)	0.150 (1.102)	(− 2.010, 2.309)
Working at start of study (yes vs no)	− 1.329 (1.293)	(− 3.862, 1.205)
LEDD	0.002 (0.002)	(− 0.002, 0.006)

### Joint evolution of apathy and cognition

**Fig. 4:**
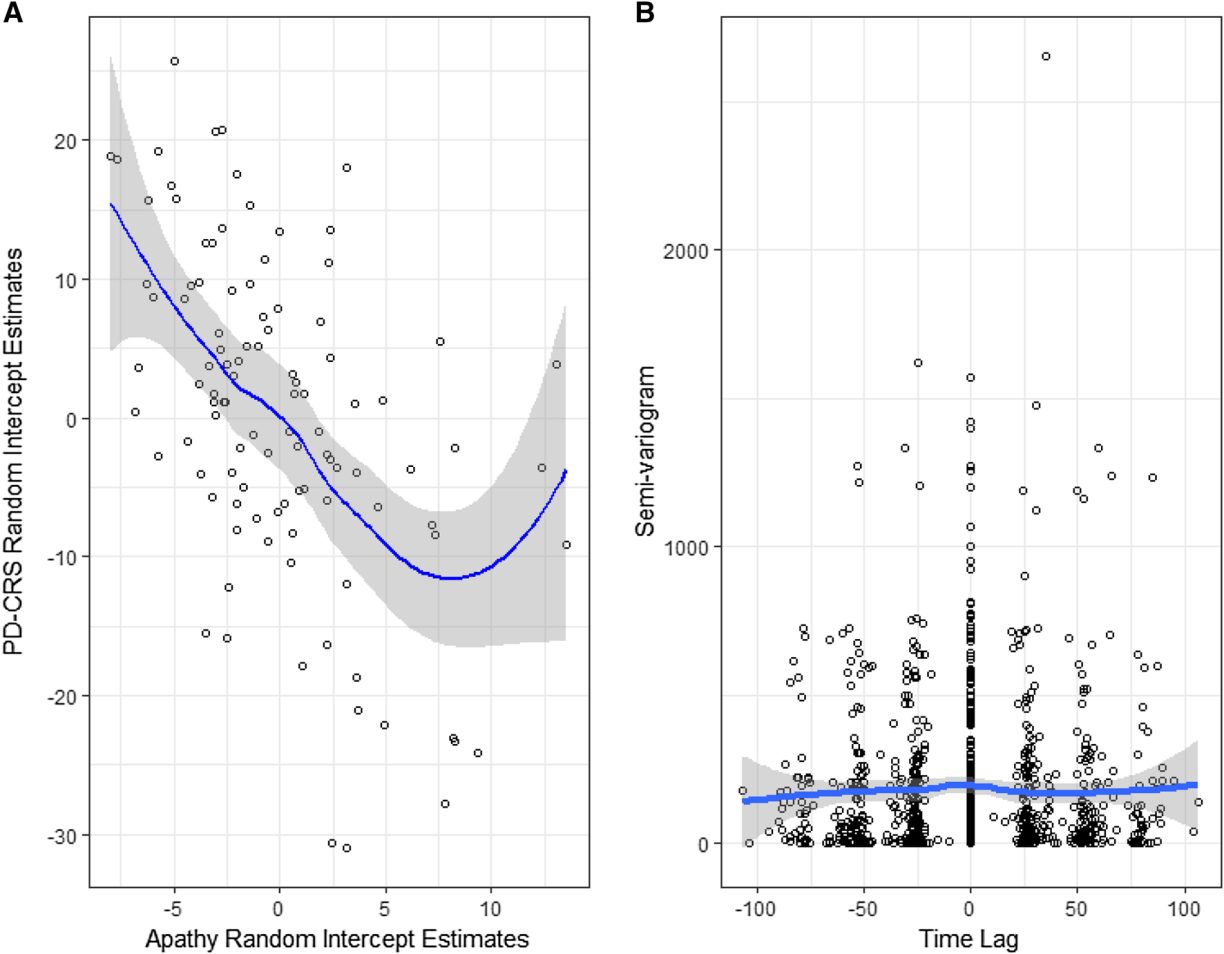
Scatter plot of the best linear unbiased predictors in the random intercepts of PD-CRS and Apathy (**a**) and the cross-empirical variogram between Apathy and PD-CRS (**b**)

We explored the best linear unbiased predictors of the random intercepts from the univariate models for self-rated Apathy Scale and the PD-CRS total scores, as exploratory analysis for potential relationships between the two outcomes. We found a statistically significant negative correlation between the random intercepts of self-rated Apathy Scale and the PD-CRS total scores (− 0.51, 95% CI − 0.64, − 0.35) (Fig. 4a). Additionally, the cross-empirical variogram between the residuals from an ordinary least squares model for the mean response of the Apathy Scale and of the PD-CRS total revealed, at best, a small lead-lag relationship (Fig. 4b). Such findings drove the choice of the functional form of the distributed lag model (see the online supplementary methods for specific details).

Specifically, we found that a joint longitudinal model between Apathy Scale and PD-CRS total scores with correlated random intercepts provided a better fit to these data than modelling the responses independently (likelihood ratio test: *p* value < 0.001). The results of the correlated random intercept joint model for Apathy Scale and PD-CRS total scores are given in Table 4. Participants with a higher (more impaired) than average self-rated Apathy Scale correlated significantly with lower (more impaired) than average PD-CRS scores (− 0.598, 95% CI − 0.744, − 0.430). We obtained similar results in our sensitivity analyses that considered PD-CRS subcortical and PD-CRS cortical domains. Here, we found that the correlation between random intercepts of the Apathy Scale and PD-CRS cortical scale scores was − 0.422 (95% CI − 0.684, − 0.117), and that between the Apathy Scale and PD-CRS subcortical scale scores was − 0.603 (95% CI − 0.749, − 0.434) (Supplementary Table 4).

**Table 4:** Joint model with correlated random intercepts between the self-rated Apathy Scale and PD-CRS total

Apathy variables	Estimate (SE)	95% CI
Intercept	11.20 (1.667)	(7.931, 14.47)
Follow-up time (weeks)	0.003 (0.007)	(− 0.012, 0.017)
Follow-up time × mean-centred baseline age	0.003 (0.001)	(0.002, 0.004)
Mean-centred baseline age (years)	0.017 (0.065)	(− 0.111, 0.146)
Disease duration at first visit (years)	− 0.082 (0.174)	(− 0.424, 0.259)
Gender (male vs. female)	− 0.259 (1.113)	(− 2.441, 1.922)
HY Score (< 2 vs 2)	0.154 (1.111)	(− 2.202, 2.332)
Working at start of study (yes vs no)	− 1.411 (1.304)	(− 3.966, 1.144)
LEDD	0.002 (0.002)	(− 0.002, 0.006)
*PD-CRS variables*		
Intercept	85.01 (4.376)	(76.43, 93.58)
Follow-up time (weeks)	0.005 (0.019)	(− 0.032, 0.042)
Follow-up time × mean-centred baseline age	− 0.002 (0.002)	(− 0.005, 0.002)
Mean-centred baseline age (years)	− 0.774 (0.171)	(− 1.110, − 0.437)
Disease duration at first visit (years)	0.501 (0.457)	(− 0.396, 1.397)
Gender (male vs. female)	− 2.393 (2.922)	(− 8.120, 3.333)
HY Score (< 2 vs 2)	0.966 (2.917)	(− 4.570, 6.683)
Working at start of study (yes vs no)	0.933 (3.422)	(− 5.774, 7.640)
LEDD	− 0.000 (0.005)	(− 0.010, 0.010)
*Cross-correlation (correlated random intercept)*		
Random intercept cross-outcome correlation	− 0.598 (0.101)	(− 0.744, -0.430)

Additionally, we fitted a joint model between the NPI apathy sub-score (binary indication of frequency × severity > 0) and PD-CRS total scores with correlated random intercepts (supplementary methods). Here, we again found that participants with a higher than average risk of scoring NPI apathy > 0 had a lower (more impaired) than average PD-CRS total, with a posterior median of the correlation between the random intercepts of − 0.665 (95% posterior credible interval: − 0.747, − 0.576).

The distributed lag model (Supplementary Table 3) did not provide a significant improvement in model fit over the correlated random intercept models (*p* = 0.995). In other words, while we found that more impaired apathy predicted more impaired cognition at any point in time (Table 4), we found no evidence to suggest that a participant’s historic self-rated Apathy Scale could predict future values of their PD-CRS total.

All these results were quantitatively similar in the sensitivity analysis that excluded all observations with PD-CRS total less than 65. The results of this sensitivity analysis are available upon request.

## Discussion

Our application of linear mixed effects methods to model the longitudinal profile of cognitive and behavioural syndromes in a cohort of people with PD revealed that cognitive and apathy scores vary widely among participants, that apathy changes minimally over time, and that there exists a clear negative correlation between cognition and apathy. Moreover, apathy and cognition appear to ‘travel together’ as reflected by departures from the mean scores in apathy ratings at any point in time being mirrored by a corresponding shift in global cognitive scores. Importantly, these linked shifts seem to occur throughout the prospective course of the condition. Finally, age of onset of PD symptoms is strongly associated with higher apathy and lower cognition ratings, further supporting the link between the syndromes.

Previous longitudinal evidence of the relationship between apathy and cognition is limited and differs in methodological approach. The advantages of our study are: (1) we directly modelled co-occurring deviations in apathy and cognition and found that worse apathy co-occurred with worse cognition, regardless of follow-up time, (2) we explored the possibility of lead-lag relationships to see if impaired apathy today could predict future cognitive decline and did not find statistically significant evidence of such a relationship, (3) we considered a bespoke model specifically designed for our hypothesis, rather than using ‘off-the-shelf’ approaches. Importantly, previous research [77, 88] did not exploit fully the longitudinal structure of the data. For example, Schrag et al. [77] examined the change in score between baseline and two-year follow-up, but did not explicitly look at time as a continuous variable. Simuni et al. [80] performed longitudinal modelling with their five-year longitudinal data, but only explored simple correlations among the outcomes, rather than jointly modelling apathy and cognition.

Apathy is comprised of multiple dimensions; cognitive, including diminished interest in new experiences and learning; behavioural, including diminished initiative and drive; diminished emotional responsivity and social interaction [66, 72]. Hence, the syndrome spans the ‘cognitive-behavioural’ divide. There has been a long debate about whether it should be classified as a separate syndrome or part of affective or cognitive disorders [82]. Our findings support the latter notion and extend the understanding of the cognitive basis of apathy.

We, and others, have previously demonstrated in cross-sectional studies that in PD, apathy is strongly associated with impairment in the executive domain [17, 23, 51–54, 62, 63, 74, 75]. However, longitudinal relationships between apathy and cognition have not previously been modelled. Devos et al. [20] followed people with newly diagnosed, untreated PD and healthy controls for 24 months as part of the PPI study. Apathy was more frequent in the PD group, with presence of apathy increasing from 16.7% to 30.2%. The severity of apathy was not investigated and apathy was omitted as one of the predictors of cognitive decline. Similarly, as far as we are aware, there are no reports about the relationship between apathy and cognition in other longitudinal early stage PD cohorts [37, 93] or parkinsonism/dementia cohorts [68]. Santangelo et al. [75] followed people with a recent diagnosis of PD for 2 years and found that, while cognition deteriorated in all participants, it was worse in those presenting with apathy at baseline. Poorer executive performance identified those that went on to develop apathy by follow-up, compared to those that did not, and this finding was supported by regression analysis.

A growing body of evidence implicates fronto-striatal dysfunction as a mechanism to explain the relationship between cognition and the apathy syndrome in Parkinson’s disease. However, apathy has also been linked to poorer cognition in multiple domains [23] and is associated with increased cortical amyloidopathy in PD [13], mirroring the findings with psychosis as a predictor of cognitive decline in PD (ffytche, Pereira et al., 2017). Our research supports the notion of apathy as a biomarker for poor cognition in PD, although at this point we cannot comment on the sensitivity or specificity of such a biomarker. However, while the correlation is stronger with the fronto-subcortical component of the cognitive assessment, apathy still correlates with the posterior-cortical component. Also, more severe apathy accompanying poorer cognition lends support to the possibility of a more intricate relationship than the syndrome being a biomarker of executive dysfunction alone.

There are several possible reasons why apathy might be associated with age. Irrespective of disorder, apathy is consistently associated with changes to the frontostriatal circuits (the dorsal anterior cingulate cortex and ventral striatum) and connecting structures (orbitofrontal cortex, ventral pallidum and ventral tegmental area) [46, 47]. In older people without dementia, apathy has previously been linked to a reduction in fronto-temporal grey and parietal/thalamic white matter volume and white matter lesions in the frontal lobe [32]. Atrophy and cerebrovascular insults acquired with age may be comorbid to or a consequence of the PD pathology. In Alzheimer’s disease, apathy correlates with the inflammatory marker, cytokine receptor IL-1RII [36]. Given that increased age is associated with a low level of chronic inflammation [67], proinflammatory cytokines are associated with lower MMSE in PD [92] and that the success in treating PD apathy with the acetylcholinesterase inhibitor rivastigmine [20] may have had an anti-inflammatory basis [84], it is possible that there might be an inflammatory mechanism behind the age-associated increase in apathy.

Accumulating evidence indicates that the role of dopamine in PD apathy is unlikely. By using an effort-based decision-making task, Le Heron et al. [46, 47] demonstrated the association between apathy and the rejection of low-reward offers. In contrast, and unrelated to levels of apathy, dopamine was associated with more responses to high effort, high reward offers on the task. While disruption to decision-making was apparent, dopamine depletion was not the cause. This is supported by PET research implicating serotonergic changes in the ventral striatum, dorsal and subgenual anterior cingulate cortices and orbitofrontal cortices in PD with apathy, compared to PD without apathy [58] and SPECT research demonstrating an association between apathy and serotonergic depletion in the dorsal raphe nucleus but no correlation between apathy and dopaminergic signal [12]. In addition to non-dopaminergic therapeutic options being most suitable for PD apathy, the growing evidence to support age-related changes in serotonergic neurotransmission, transporter and receptor expression [73] and serotonergic association with cortical amyloidopathy in PD [44] may also provide plausible explanations for the mechanisms behind age-related risk of worse apathy.

Our study is limited by its relatively small sample size. Ideally, a cohort would be recruited as de novo, untreated participants with PD and followed for longer than 24 months. We did not examine the relationship between cognition and fatigue and it would be wise to do so in future research. Here, we did not observe a decline in cognition, despite a proportion of the participants classified as having PDD. As MMSE scores only reduce by 0.2–4.5 points per year, we would not expect cognition to decline much over the two year follow-up period [14, 91]. However, to be certain, any future research should use a battery of cognitive tasks, in addition to the PD-CRS. The models presented in this study, while mathematically complex, simply represent the vehicle that has allowed us to investigate our original hypothesis. Finally, it should be acknowledged that since the development of the tools with which we measure apathy [59, 60, 83], our understanding of the syndrome and the diagnostic criteria [72] have undergone substantial change. Ideally, we need to see comparable revision of the measures we use, so that they may accommodate all aspects of apathy.

## Conclusion

Our evidence further supports the link between cognition and apathy. While increased apathy may not predict future levels of cognitive performance, current presence of apathy is a behavioural indicator of cognitive impairment, and more severe apathy is anchored to poorer cognitive function. In crossing the behavioural-cognitive divide, apathy provides clinicians with a means to identify those who require additional support for cognitive problems within the clinical setting, adding to the armamentarium. Further longitudinal research with brain imaging is required to increase understanding of brain mechanisms behind apathy and cognition, but also the related association with increased age.

## Electronic supplementary material

Below is the link to the electronic supplementary material.

Supplementary material 1 (DOCX 97 kb)
