# Supplementary material for: Apathy as a behavioural marker of cognitive impairment in Parkinson’s disease: a longitudinal analysis
Source: J Neurol. 2019 Oct 15;267(1):214–27. doi: 10.1007/s00415-019-09538-z (PMC6954881; doi:10.1007/s00415-019-09538-z)
Supplement: Supplementary file 1 — Supplementary material 1 (DOCX 97 kb) [file 415_2019_9538_MOESM1_ESM.docx]

# Online Supplementary Material

## Supplementary Methods

Let $Y_{i,j,1}$ and $Y_{i,j,2}$ denote the $j$-th measurement of self-rated Apathy Scale and PD-CRS, respectively, on the $i$-th participant, for a subject-specific follow-up time $t_{i,j}$ (representing weeks since time zero). Moreover, write $\boldsymbol{X}_{ij}$ to be the $1\times P$ design matrix of covariates, which includes follow-up time, mean-centred baseline age (at time zero), disease duration at first visit, gender, HY score, indication of working status (at time zero), LEDD, and an interaction term between baseline age and follow-up time. Within this structure, we use a class of linear mixed effects models of the form

$$Y_{i,j,k}=\boldsymbol{X}_{ij}\boldsymbol{\beta}_{k}+U_{i,k}+W_{i,k}\left( t_{i,j} \right)+Z_{i,j,k}:j=1,\ldots,m_{i};i=1,\ldots,N;k=1,2; (1)$$

where $\boldsymbol{\beta}_{k}$ is the vector of fixed effect coefficients, $U_{i,k}\sim N(0,\omega_{k}^{2})$ are independent random intercepts, $Z_{i,j,k}\sim N(0,\tau_{k}^{2})$ are independent measurement errors, and ${\{W}_{i,k}\left( t_{i,j} \right)\}$ are a set of independent realisations from a zero-mean Gaussian stochastic processes with covariance function $\sigma_{k}^{2}r_{k}(t_{i,j}-t_{i,j^{'}})$. We used the exponential correlation function: $r_{k}\left( u \right)=\exp(-|u|/\phi_{k})$.

Our primary hypothesis was that the apathy could be a predictor of cognitive function. To investigate this, we adopted a multivariate model that allowed for correlation between the random components of equation (1). Such an approach was motivated by examining visual relationships between the best linear unbiased predictors from each univariate model of self-rated Apathy Scale and PD-CRS. Specifically, let $\boldsymbol{J}_{i,k}$ denote the $m_{i}\times m_{i}$ matrix, with the $\left( j,j^{'} \right)$^th^ element as the correlation between $W_{i,k}\left( t_{i,j} \right)$ and $W_{i,k}\left( t_{i,j'} \right)$, $\boldsymbol{I}_{\boldsymbol{m}_{\boldsymbol{i}}}$ denote the $m_{i}\times m_{i}$ identity matrix and $\boldsymbol{1}_{\boldsymbol{m}_{\boldsymbol{i}}}$ denote the $m_{i}\times m_{i}$ matrix with each element as one. Then, for each response $k$, we have

$$\left[ \begin{matrix} \boldsymbol{Y}_{\boldsymbol{i,k}} \\ \boldsymbol{U}_{\boldsymbol{i,k}} \\ \boldsymbol{W}_{\boldsymbol{i,k}}(\boldsymbol{t}_{i}) \end{matrix} \right]\sim MVN\left[ \left( \begin{matrix} \boldsymbol{X}_{ij}\boldsymbol{\beta}_{k} \\ \boldsymbol{0} \\ \boldsymbol{0} \end{matrix} \right),\left( \begin{matrix} \Sigma_{Y_{k}} & \Sigma_{UY_{k}} & \Sigma_{WY_{k}} \\ \Sigma_{UY_{k}} & \Sigma_{U_{k}} & \Sigma_{UW_{k}} \\ \Sigma_{WY_{k}} & \Sigma_{UW_{k}} & \Sigma_{W_{k}} \end{matrix} \right) \right],$$

where $\Sigma_{Y_{k}}=\omega_{k}^{2}\boldsymbol{1}_{\boldsymbol{m}_{\boldsymbol{i}}}+\sigma_{k}^{2}\boldsymbol{J}_{i,k}+\tau_{k}^{2}\boldsymbol{I}_{\boldsymbol{m}_{\boldsymbol{i}}}$, $\Sigma_{U_{k}}=\Sigma_{UY_{k}}=\omega_{k}^{2}\boldsymbol{1}_{\boldsymbol{m}_{\boldsymbol{i}}}$, $\Sigma_{W_{k}}=\Sigma_{WY_{k}}=\sigma_{k}^{2}\boldsymbol{J}_{i,k}$ and $\Sigma_{UW_{k}}=\boldsymbol{0}$. Thus, the best linear unbiased predictors for the random intercept and serial correlation terms are

$$E\left[ U_{i,k} | Y_{i,k} \right]=\Sigma_{UY_{k}}\Sigma_{Y_{k}}^{-1}(\boldsymbol{Y}_{i,k}-\boldsymbol{\mu}_{i,k})$$

$$E\left[ W_{i,k} | Y_{i,k} \right]=\Sigma_{WY_{k}}\Sigma_{Y_{k}}^{-1}\left( \boldsymbol{Y}_{i,k}-\boldsymbol{\mu}_{i,k} \right).$$

Based on these, we considered two multivariate modelling cases. Firstly, we assumed correlated random intercepts as $(U_{i,1},U_{i,2})\sim MVN(\boldsymbol{0}, \Sigma_{U})$, where $\Sigma_{U}$ had diagonal elements $\omega_{1}^{2}$ and $\omega_{2}^{2}$ and off-diagonal elements $\omega_{1}\omega_{2}\rho_{U}$. The second multivariate model again assumed that $\left( U_{i,1},U_{i,2} \right)$ were correlated, but we additionally imposed a distributed lag model with directed dependence between the serial correlation terms. Specifically, since our *a priori* assumption was that self-rated Apathy Scale could be used to predict PD-CRS, the dependence between ${\{W}_{i,1}\left( t \right)\}$ and $\{W_{i,2}\left( t \right)\}$ was defined by

$$W_{i,2}\left( t_{i,j} \right)=E_{i,j}+\int_{0}^{\infty} \alpha\left( v \right)W_{i,1}\left( t_{i,j}-v \right)dv,$$

where $E_{i,j}\sim N(0,\nu^{2})$ and the form of the function $\alpha\left( v \right)$ was specified by examining the cross-correlation structure between pairs of responses. Hence, we have that

$$\left[ \begin{matrix} \boldsymbol{Y}_{i,1} \\ \boldsymbol{Y}_{i,2} \end{matrix} \right]\sim\mathrm{MVN}\left( \left[ \begin{matrix} \boldsymbol{X}_{i}\boldsymbol{\beta}_{1} \\ \boldsymbol{X}_{i}\boldsymbol{\beta}_{2} \end{matrix} \right],\left[ \begin{matrix} \Sigma_{Y_{1},i} & \Sigma_{Y_{1}Y_{2},i} \\ \Sigma_{Y_{1}Y_{2},i} & \Sigma_{Y_{2},i} \end{matrix} \right] \right), (2)$$

with the form of the variance-covariance matrices dependent on modelling assumption. For the first multivariate modelling assumption, we have that $\Sigma_{Y_{1}Y_{2},i}=\omega_{1}\omega_{2}\rho_{u}\boldsymbol{1}_{\boldsymbol{m}_{\boldsymbol{i}}}$. For the second assumption, we had to derive the auto- and cross-covariance of $\{W_{i,1}\left( t \right),W_{i,2}\left( t \right)\}$. Here, write $\sigma_{k}^{2}=\mathrm{Var}\left\{ W_{i,k}\left( t \right) \right\}$ and $r_{kl}\left( u \right)=\mathrm{Corr}\{W_{i,k}\left( t \right), W_{i,l}\left( t-u \right)\}$, then it follows that

$$\mathrm{Var}\left\{ W_{i,2}\left( t \right) \right\}=\nu^{2}+\sigma_{1}^{2}\int_{0}^{\infty} \int_{0}^{\infty} \alpha\left( v \right)\alpha\left( v^{'} \right)r_{11}\left( v^{'}-v \right)dvdv'.$$

Limiting $\alpha\left( v \right)$ to have only finite support automatically ensures that the above integral is finite. Subject to this condition, and for $u\neq0$, the auto- and cross-covariance functions are given by

$$\mathrm{Cov}\left\{ W_{i,2}\left( t \right),W_{i,2}\left( t-u \right) \right\}=\sigma_{1}^{2}\int_{0}^{\infty} \int_{0}^{\infty} \alpha\left( v \right)\alpha\left( v^{'} \right)r_{11}\left( u+v^{'}-v \right)dvdv'$$

$$\mathrm{Cov}\left\{ W_{i,2}\left( t \right),W_{i,1}\left( t-u \right) \right\}=\sigma_{1}^{2}\int_{0}^{\infty} \alpha\left( v \right)r_{11}\left( u-v \right)dv$$

Hence, the covariance structure of equation (2) for the distributed lag model (the second multivariate modelling assumption) can be defined by the following:

$$\mathrm{Var}\left\{ Y_{i,j,1} \right\}=\omega_{1}^{2}+\sigma_{1}^{2}+\tau_{1}^{2}$$

$$\mathrm{Cov}\left\{ Y_{i,j,1},Y_{i,j^{'},1} \right\}=\omega_{1}^{2}+\sigma_{1}^{2}exp(-|t_{ij}-t_{i,j^{'}}|/\phi_{1})$$

$$\mathrm{Var}\left\{ Y_{i,j,2} \right\}=\omega_{2}^{2}+\mathrm{Var}\left\{ W_{i,2}\left( t \right) \right\}+\tau_{2}^{2}$$

$$\mathrm{Cov}\left\{ Y_{i,j,2},Y_{i,j^{'},2} \right\}=\omega_{2}^{2}+\mathrm{Cov}\left\{ W_{i,2}\left( t_{ij} \right),W_{i,2}\left( t_{ij}-u \right) \right\};u=t_{ij}-t_{ij'}$$

$$\mathrm{Cov}\left\{ Y_{ij1},Y_{i,j^{'},2} \right\}=\omega_{1}\omega_{2}\rho_{u}+\mathrm{Cov}\left\{ W_{i,2}\left( t_{ij} \right),W_{i,1}\left( t_{ij}-u \right) \right\};u=t_{ij}-t_{ij'}$$

In the current study, exploratory analysis revealed a very small lead-lag relationship between self-rated Apathy and PD-CRS. Thus, we defined $\alpha\left( v \right)$ to be

$$\alpha\left( v \right)=\left\{ \begin{matrix} \psi_{1} & \mathrm{if}0<v\leq\psi_{2} \\ 0 & \mathrm{Otherwise} \end{matrix} \right.$$

from which the auto- and cross-covariance structure of the distributed lag model can be obtained analytically. Inference for both the correlated random intercepts model and the distributed lag model was undertaken through R code written by the authors.

For the multivariate models that aimed to explore the association between NPI apathy sub-score (binary indicator of frequency$\times$severity>0) and PD-CRS total, we again assumed a correlation between the random intercepts. Explicitly, if $Y_{i,j,3}\sim Binomial(\pi_{ij})$, where $Y_{i,j,3}$ denotes the binary indicator of NPI apathy sub-score >0 and $\pi_{ij}=P(Y_{i,j,3}=1)$, then we modelled a corresponding version of equation (2) as:

$$\log\left( \frac{\pi_{ij}}{1-\pi_{ij}} \right)=\boldsymbol{X}_{ij}\boldsymbol{\beta}_{3}+U_{i,3}+W_{i,3}\left( t_{i,j} \right)$$

$$Y_{i,j,2}=\boldsymbol{X}_{ij}\boldsymbol{\beta}_{2}+U_{i,2}+W_{i,2}\left( t_{i,j} \right)+Z_{i,j,2}$$

where $U_{i,3}\sim N(0,\omega_{3}^{2})$ is a random intercept and $\mathrm{Corr}\left\{ U_{i,3},U_{i,2} \right\}=\rho_{U_{3}U_{2}}$. As above, ${\{W}_{i,3}\left( t_{i,j} \right)\}$ are a set of independent realisations from a zero-mean Gaussian stochastic processes with exponential correlation function. Due to the intractability of a joint Binomial and Gaussian likelihood, the high-dimensional integral over the random effects was approximated with Markov Chain Monte Carlo (MCMC). This was undertaken through user written code in the ‘rstan’ R package. Weakly informative Gaussian prior distributions were placed on the fixed effect coefficients and an inverse-Gamma distributions on the random effect covariance structures and hyper-parameters.

## Supplementary Figures


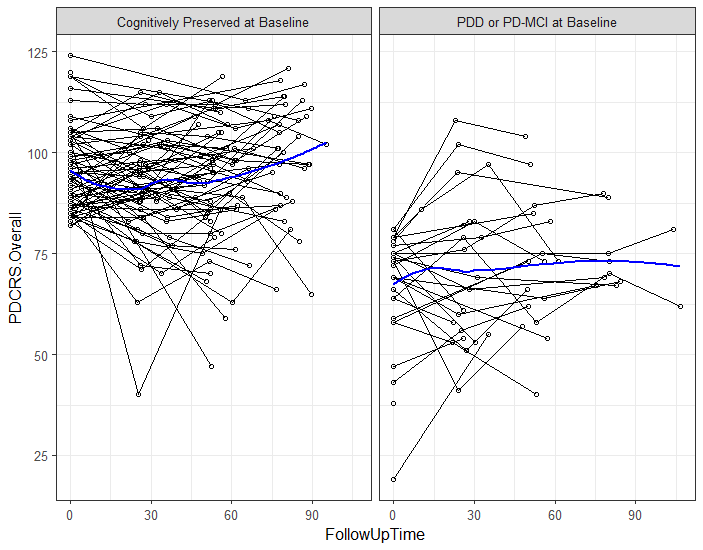


**Supplementary Figure 1**: Time-plot of PD-CRS total score, stratified by those with a PD-CRS < 82 (PDD or PD-MCI) against those with PD-CRS > 82 (cognitively preserved) at baseline


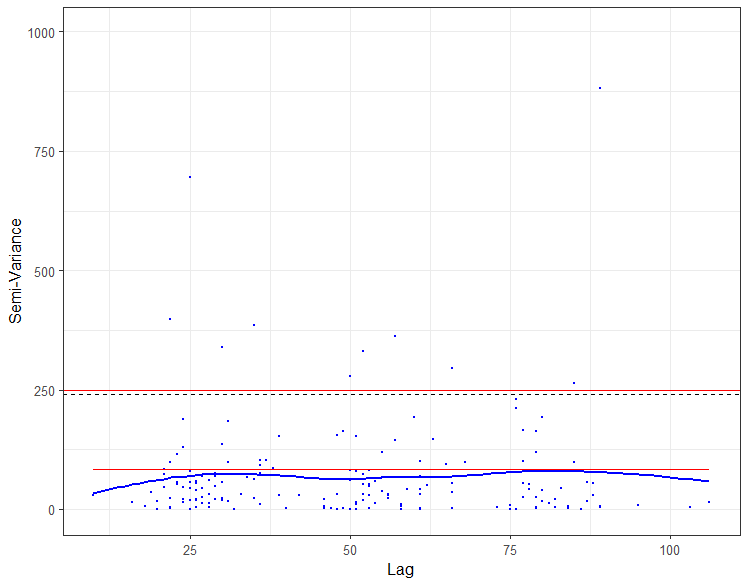


**Supplementary Figure 2**: Empirical and fitted variogram for the univariate model of PD-CRS Total


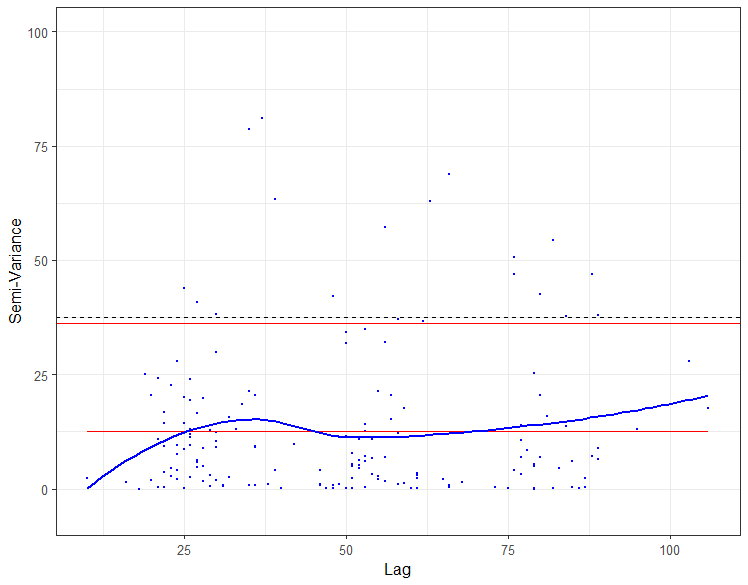


**Supplementary Figure 3**: Empirical and fitted variogram for the univariate model of self-rated Apathy Scale

## Supplementary Tables

**Supplementary Table 1:** Clinical characteristics of participants with PD in the cohort at each participant’s final visit (n=104)

| **Variables (as recorded at each participant’s last visit)** | | **Summary** |
| --- | --- | --- |
| Cognition | MMSE, median (IQR) [min, max] | 29 (27, 30) [19, 30] |
|  | PD-CRS Total Score, median (IQR) [min, max] | 88 (73, 101.3) [0, 121] |
|  | PD-CRS Subcortical, median (IQR) [min, max] | 59.5 (45, 72) [0, 91] |
|  | PD-CRS Cortical, median (IQR) [min, max] | 29 (28, 30) [0, 30] |
| Function | Schwab-England score, median (IQR) [min, max] | 80 (70, 90) [40, 100] |
|  | EQ-VAS, median (IQR) [min, max] | 78.5 (60, 84.3) [20, 98] |
|  | EQ-5D, median (IQR) [min, max] | 7.5 (6, 8) [5, 11] |
|  | PDQ-39 Domain 1, median (IQR) [min, max] | 9 (3, 16) [0, 36] |
|  | PDQ-39 Domain 2, median (IQR) [min, max] | 5 (2, 8.25) [0, 14] |
|  | PDQ-39 Domain 3, median (IQR) [min, max] | 3 (1, 6) [0, 16] |
|  | PDQ-39 Domain 4, median (IQR) [min, max] | 1 (0, 3.25) [0, 15] |
|  | PDQ-39 Domain 5, median (IQR) [min, max] | 0 (0, 1) [0, 8] |
|  | PDQ-39 Domain 6, median (IQR) [min, max] | 4 (2, 5) [0, 10] |
|  | PDQ-39 Domain 7, median (IQR) [min, max] | 1 (0, 2) [0, 6] |
|  | PDQ-39 Domain 8, median (IQR) [min, max] | 3 (1, 6) [0, 10] |
| Motor Score | UPDRS, median (IQR) [min, max] | 51 (33.8, 70.5) [12, 114] |
| Neuropsychiatric symptoms | NPI total*, median (IQR) [min, max] | 3 (0.75, 7.25) [0, 59] |
|  | NPI total* ≥4, n (%) | 50 (48.1%) |
|  | NPI total* >0, n (%) | 78 (75.0%) |
|  | HADS anxiety, median (IQR) [min, max] | 5 (3, 8) [0, 13] |
|  | HADS depression, median (IQR) [min, max] | 4 (2, 6) [0, 17] |
| Apathy | Apathy self-rated, median (IQR) [min, max] | 10.5 (6, 15) [0, 29] |
|  | NPI apathy sub-score*, median (IQR) [min, max] | 0 (0, 0) [0,12] |
|  | NPI apathy sub-score* >0, n (%) | 23 (22.1%) |
|  | NPI apathy sub-score* ≥4, n (%) | 4 (3.85%) |
| Sleep | Epworth Sleepiness Scale, median (IQR) [min, max] | 7 (5, 11) [1, 19] |
|  | NPI sleep sub-score*, median (IQR) [min, max] | 0 (0, 2.5) [0, 12] |
|  | NPI sleep sub-score* >0, n (%) | 36 (34.6%) |
|  | NPI apathy sub-score* ≥4, n (%) | 17 (16.3%) |

*Abbreviations: PD-CRS = Addenbrooke’s Cognitive Evaluation; EQ = EuroQoL-5D index or visual analogue scale (VAS); HADS = Hospital Anxiety and Depression Scale; IQR = interquartile range; MMSE = Mini-mental State Exam; NPI = Neuropsychiatric Inventory; PDQ-39 = Parkinson’s Disease Questionnaire; UPDRS = Unified Parkinson’s Disease Rating Scale; PD = Parkinson’s Disease*

**NPI mean domain score: frequency*$\times$*severity*

**Supplementary Table 2:** Posterior medians and 95% credible intervals for the fixed effect estimates from the MCMC algorithm for the univariate analysis of NPI apathy sub-scale (binary indication of frequency$\times$severity>0), which included the random intercept and stationary Gaussian Process random effects.

| **Variable** | **Posterior Median (odds ratio)** | **95% Credible Interval (odds ratio)** |
| --- | --- | --- |
| Follow-up time (weeks) | 0.997 | (0.991, 1.003) |
| Follow-up time $\times$ Mean-Centred Baseline Age | 1.000 | (1.000, 1.001) |
| Mean-Centred Baseline Age (years) | 0.992 | (0.956, 1.028) |
| Disease duration at first visit (years) | 0.753 | (0.681, 0.824) |
| Gender (Male vs. Female) | 0.791 | (0.488, 1.365) |
| HY Score (<2 vs 2) | 0.497 | (0.302, 0.767) |
| Working at start of study (yes vs no) | 0.445 | (0.246, 0.809) |
| LEDD | 1.001 | (1.000, 1.002) |

**Supplementary Table 3:** Distributed Lag Joint Model between self-rated Apathy Scale and PD-CRS total

| **Apathy Variables** | **Estimate (SE)** | **95% CI** |
| --- | --- | --- |
| Intercept | 11.20 (1.667) | (7.931, 14.47) |
| Follow-up time (weeks) | 0.003 (0.007) | (-0.012, 0.017) |
| Follow-up time $\times$ Mean-Centred Baseline Age | 0.003 (0.001) | (0.002, 0.004) |
| Mean-Centred Baseline Age (years) | 0.017 (0.065) | (-0.111, 0.146) |
| Disease duration at first visit (years) | -0.082 (0.174) | (-0.424, 0.259) |
| Gender (Male vs. Female) | -0.259 (1.113) | (-2.441, 1.922) |
| HY Score (<2 vs 2) | 0.155 (1.111) | (-2.023, 2.332) |
| Working at start of study (yes vs no) | -1.411 (1.304) | (-3.965, 1.144) |
| LEDD | 0.002 (0.002) | (-0.002, 0.006) |
| **PD-CRS Variables** |  |  |
| Intercept | 85.01 (4.374) | (76.43, 93.58) |
| Follow-up time (weeks) | 0.005 (0.019) | (-0.032, 0.042) |
| Follow-up time $\times$ Mean-Centred Baseline Age | -0.002 (0.002) | (-0.005, 0.002) |
| Mean-Centred Baseline Age (years) | -0.774 (0.171) | (-1.110, -0.438) |
| Disease duration at first visit (years) | 0.501 (0.457) | (-0.396, 1.397) |
| Gender (Male vs. Female) | -2.393 (2.922) | (-8.118, 3.331) |
| HY Score (<2 vs 2) | 0.966 (2.915) | (-4.748, 6.681) |
| Working at start of study (yes vs no) | 0.933 (3.420) | (-5.771, 7.637) |
| LEDD | 0.000 (0.005) | (-0.010, 0.010) |
| **Cross-correlation** |  |  |
| Random intercept cross-outcome correlation | -0.598 (0.101) | (-0.743, -0.430) |
| Distributed Lag association ($\psi_{1}$) | -0.069 | NA |
| Distributed Lag range ($\psi_{2}$) | 5.529 | NA |

**Supplementary Table 4:** Multivariate analysis for PD-CRS Cortical and PD-CRS Subcortical with self-rated Apathy Scale

| **Model** | **Random intercept cross-outcome correlation** |
| --- | --- |
| PD-CRS Cortical & Apathy | -0.422 (-0.684, -0.117) |
| PD-CRS Subcortical & Apathy | -0.603 (-0.749, -0.434) |
